# Supplementary material for: Identification of two major autoantigens negatively regulating endothelial activation in Takayasu arteritis
Source: Nat Commun. 2020 Mar 9;11:1253. doi: 10.1038/s41467-020-15088-0 (PMC7062749; doi:10.1038/s41467-020-15088-0)
Supplement: Supplementary file 1 — Supplementary Information [file 41467_2020_15088_MOESM1_ESM.pdf]

Supplementary Information

Identification of two major autoantigens negatively regulating endothelial activation in Takayasu arteritis

Tomoyuki Mutoh, Tsuyoshi Shirai*, Tomonori Ishii, Yuko Shirota, Fumiyoshi Fujishima, Fumiaki Takahashi, Yoichi Kakuta, Yoshitake Kanazawa, Atsushi Masamune, Yoshikatsu Saiki, Hideo Harigae, Hiroshi Fujii

*Correspondence: T. Shirai. Email adress: tsuyoshirajp@med.tohoku.ac.jp

This file includes:

Supplementary Figure 1 to 17
Supplementary Table 1 and 2
Supplementary Method

Supplementary Figures

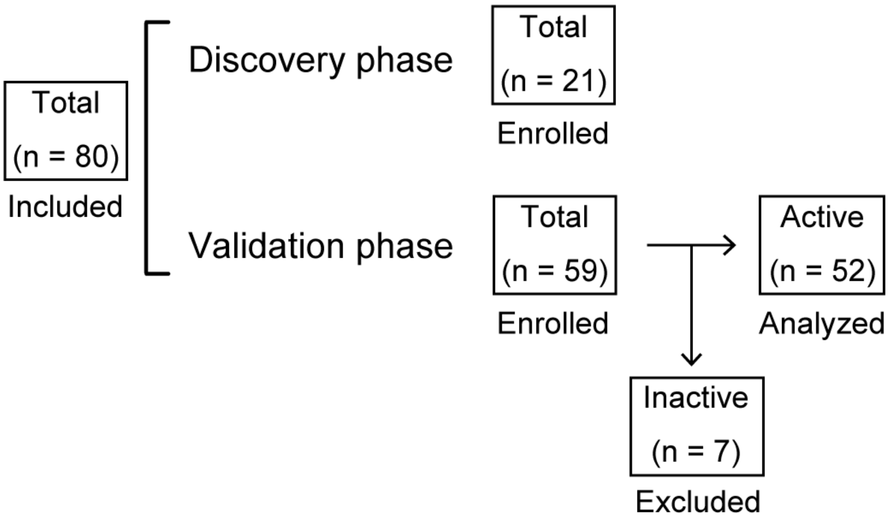

Supplementary Fig. 1 Analysis flowchart of 80 patients with Takayasu arteritis in discovery and validation phase.

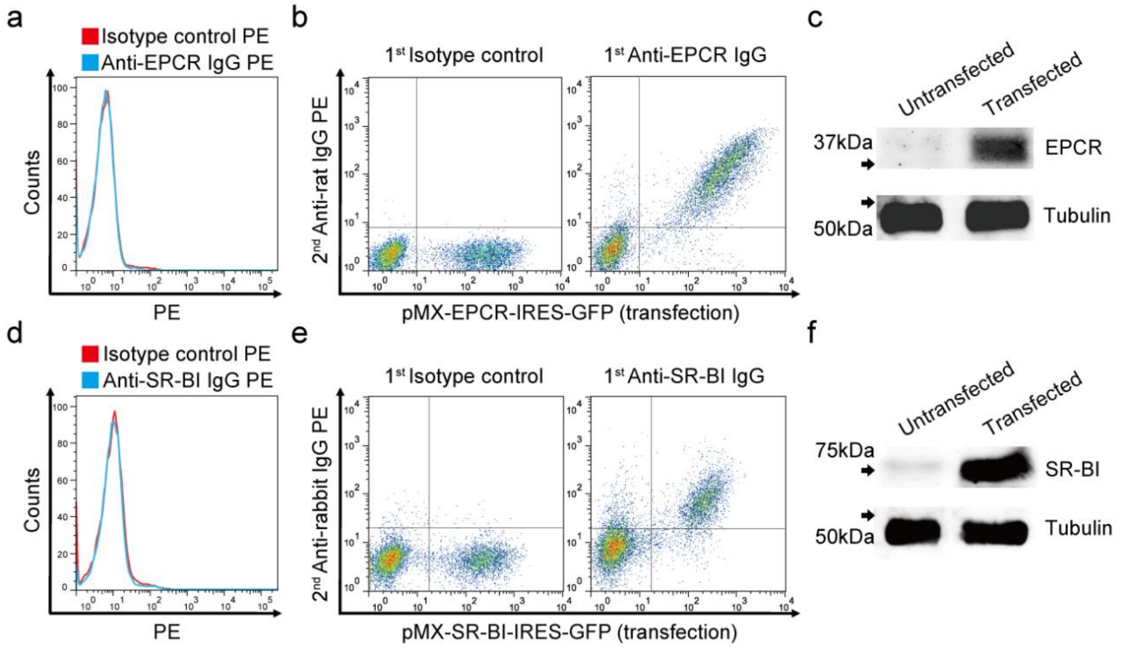

Supplementary Fig. 2 Generation of YB2/0 cells expressing endothelial protein C receptor (EPCR) or scavenger receptor class B type 1 (SR-BI).

a YB2/0 cells were stained with PE-conjugated isotype control or PE-conjugated anti-human EPCR IgG and analyzed with flow cytometry. **b** Expression vector pMX-EPCR-IRES-GFP is transfected into YB2/0 cells. The cells were stained with anti-EPCR commercial IgG or isotype control followed by PE-conjugated secondary antibody and analyzed with flow cytometry. **c** Western blotting analysis of the expression of EPCR and tubulin in untransfected or EPCR-transfected YB2/0 cells. **d** YB2/0 cells were stained with PE-conjugated isotype control or PE-conjugated anti-human SR-BI IgG and analyzed with flow cytometry. **e** Expression vector pMX-SR-BI-IRES-GFP is transfected into YB2/0 cells. The cells were stained with anti-SR-BI commercial IgG or isotype control followed by PE-conjugated secondary antibody and analyzed with flow cytometry. **f** Western blotting analysis of the expression of SR-BI and tubulin in untransfected or SR-BI-transfected YB2/0 cells.

a

Gene	Signal	Basal Signal	Log2 Ratio
PROCR	528932.6	186.0	11.47
SCARB1	61076.4	99.9	9.26
SCARB1	120463.0	1176.5	6.68

b

Activity	U10-4	W10-59	X10-11	G10-43	Z10-39	U10-53	A11-08	B11-45	C11-12
Anti-EPCR	○	—	○	—	○	—	○	—	—
Anti-SR-BI	—	○	—	○	—	○	—	○	—

Supplementary Fig. 3 Validation of EPCR and SR-BI as novel autoantigens in Takayasu arteritis.

a Microarray analysis comparing unsorted YB 2/0 cells and a mixture of cloned cells established with U10-4 IgG, W10-59 IgG, and G10-43 IgG. **b** Anti-EPCR and anti-SR-BI activities were measured among nine TAK serum samples, which were initially selected for cell sorting. Circles and lines indicate positive and negative, respectively. U10-4, W10-59, G10-43, Z10-39, U10-53, A11-08, B11-45, and C11-12 indicate the serum sample number.

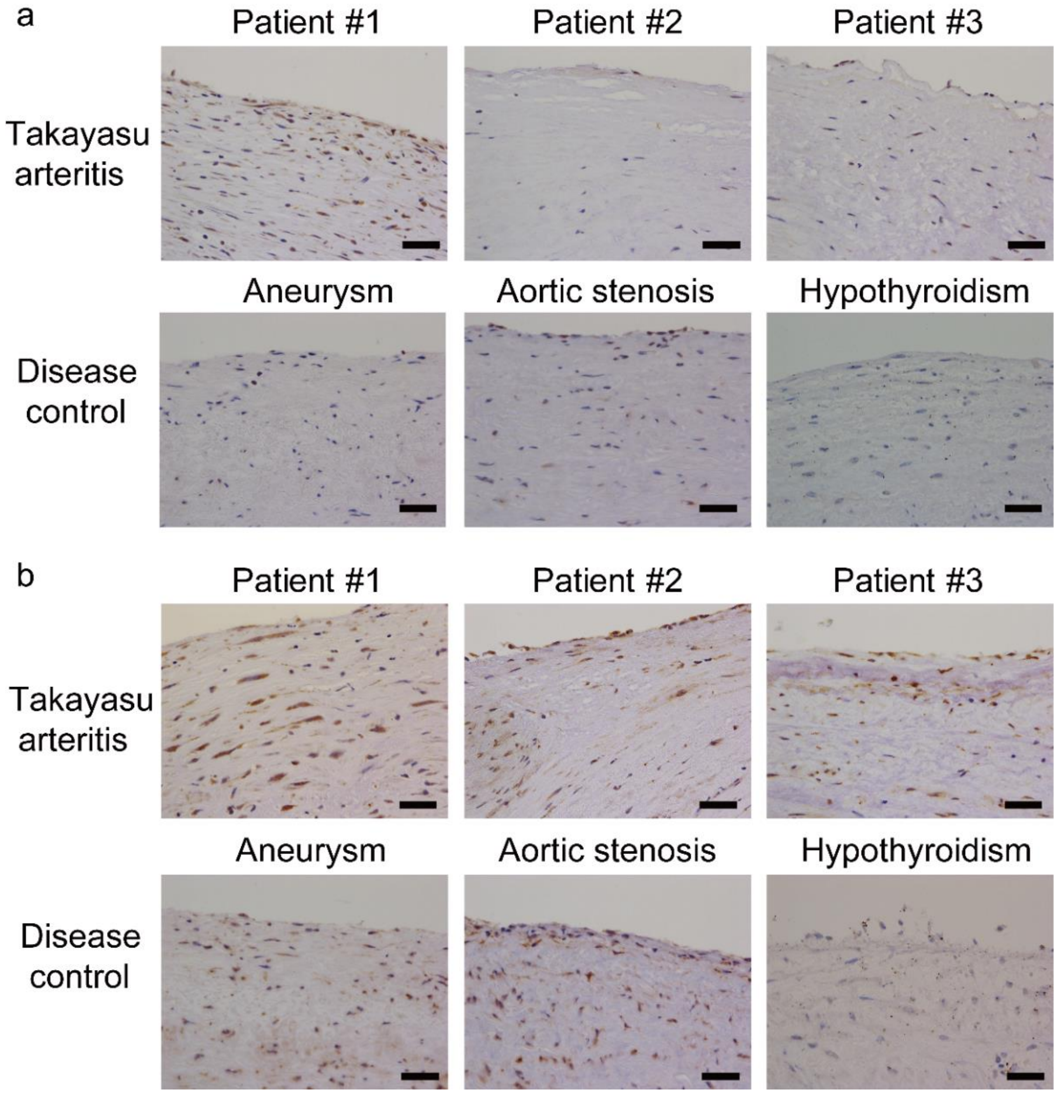

Supplementary Fig. 4 Expressions of EPCR and SR-BI in the intima of the aorta. Immunohistochemical analysis was performed against EPCR (**a**) and SR-BI (**b**) in resected aortic tissue from three different patients with TAK (upper column) and non-inflammatory aortic tissue as disease controls (lower column). Disease controls included surgical specimen from patients with aortic aneurysm and aortic stenosis, and an autopsy sample from a patient with hypothyroidism. Expressions in the intima of the aorta are shown. Black bar indicates 40 μm.

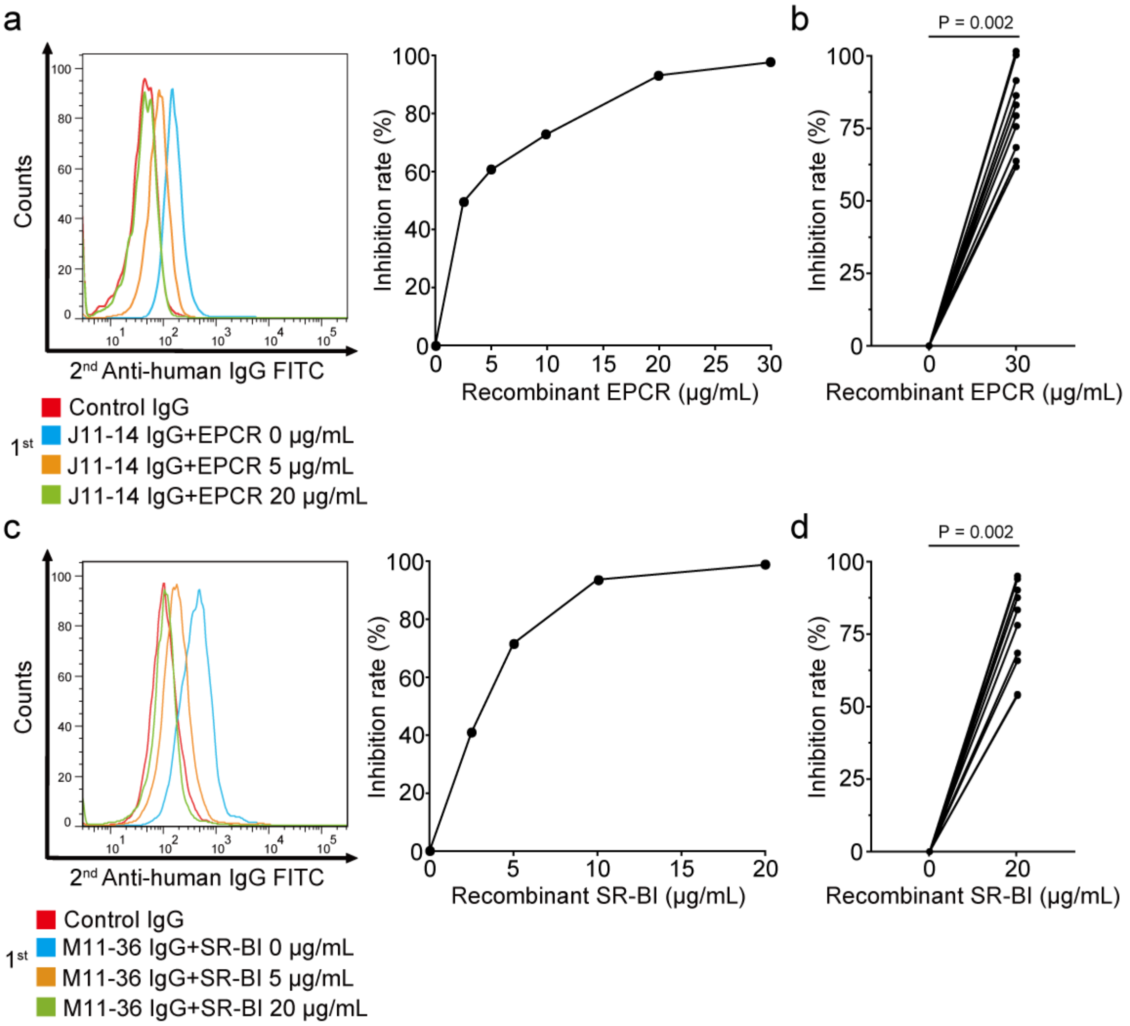

Supplementary Fig. 5 Inhibition test of AECAs activity with recombinant protein.
a Inhibition test of binding activities to human umbilical vein endothelial cells (HUVECs) was conducted using 0.5 mg/mL control IgG or IgG from anti-EPCR positive TAK AECA (J11-14) with soluble recombinant EPCR at the indicated concentrations. Representative histograms (left) and summarized graph (right) are shown. **b** Inhibition rates were measured in 10 TAK AECA with anti-EPCR activities. Statistical analysis was performed using the Wilcoxon signed-rank test. **c** Inhibition test against HUVECs was conducted using 0.5 mg/mL control IgG or IgG from anti-SR-BI positive TAK AECA (M11-36) with soluble recombinant SR-BI at the indicated concentrations. Representative histograms (left) and summarized graph (right) are shown. **d** Inhibition rates were measured in 10 TAK AECA with anti-SR-BI activities. Statistical analysis was performed using the Wilcoxon signed-rank test.

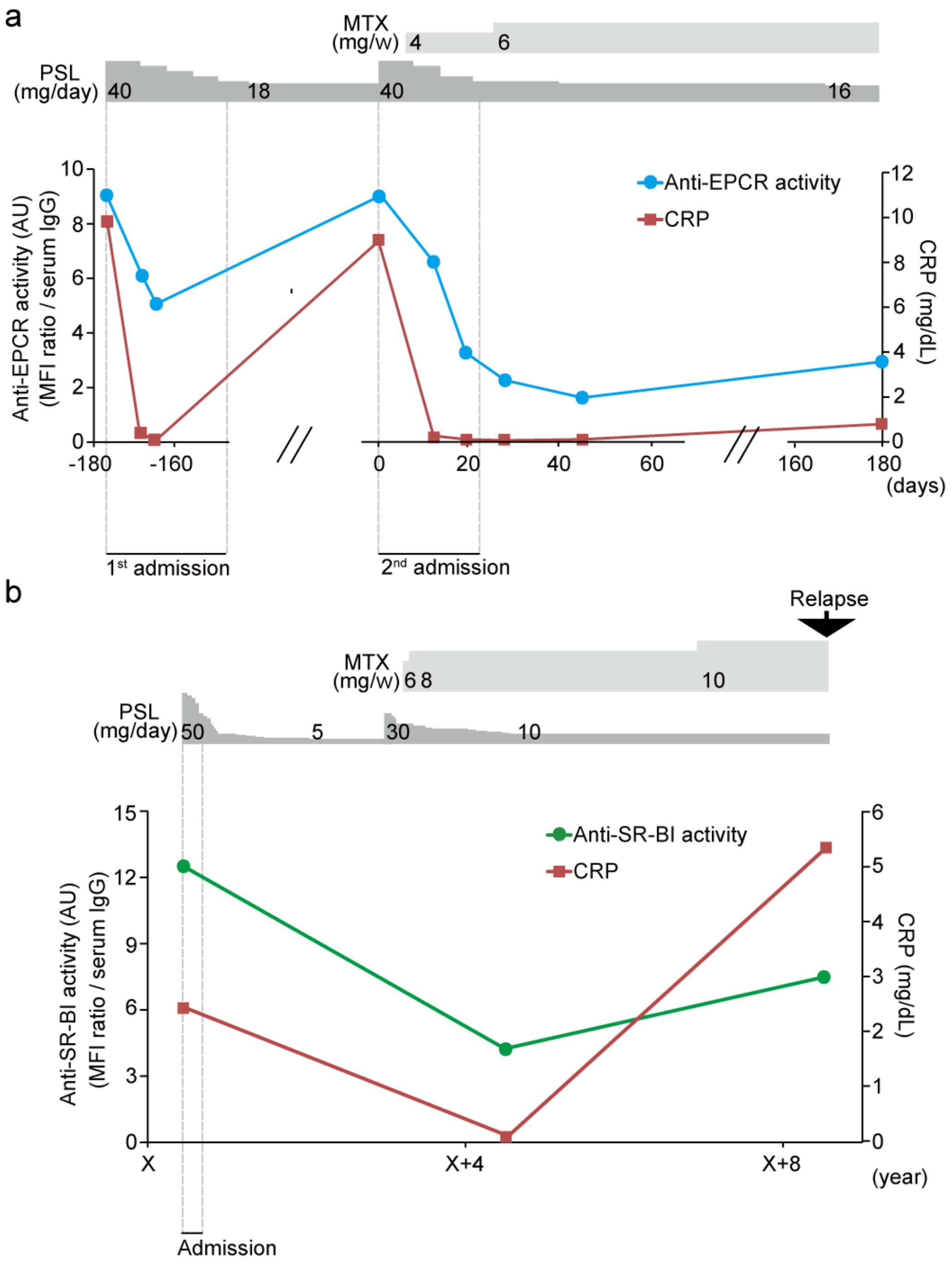

Supplementary Fig. 6 Clinical course.

a, **b** Clinical course of a TAK patient with anti-EPCR autoantibodies (**a**) or anti-SR-BI autoantibodies (**b**). AU, arbitrary unit; CRP, C-reactive protein; PSL, prednisolone; MFI, mean fluorescent intensity; MTX, methotrexate.

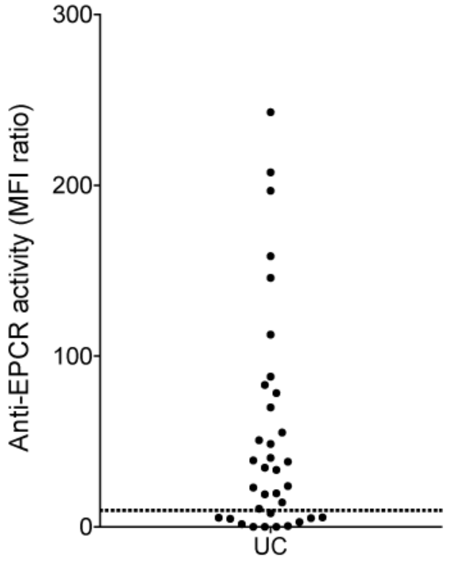

Supplementary Fig. 7 Anti-EPCR autoantibodies in primary ulcerative colitis

The distribution of anti-EPCR autoantibodies in patients with ulcerative colitis (UC, n=35). Dots represent the data for individual subjects. The broken horizontal line indicates the cut-off value for high activity (mean + 3SD of controls).

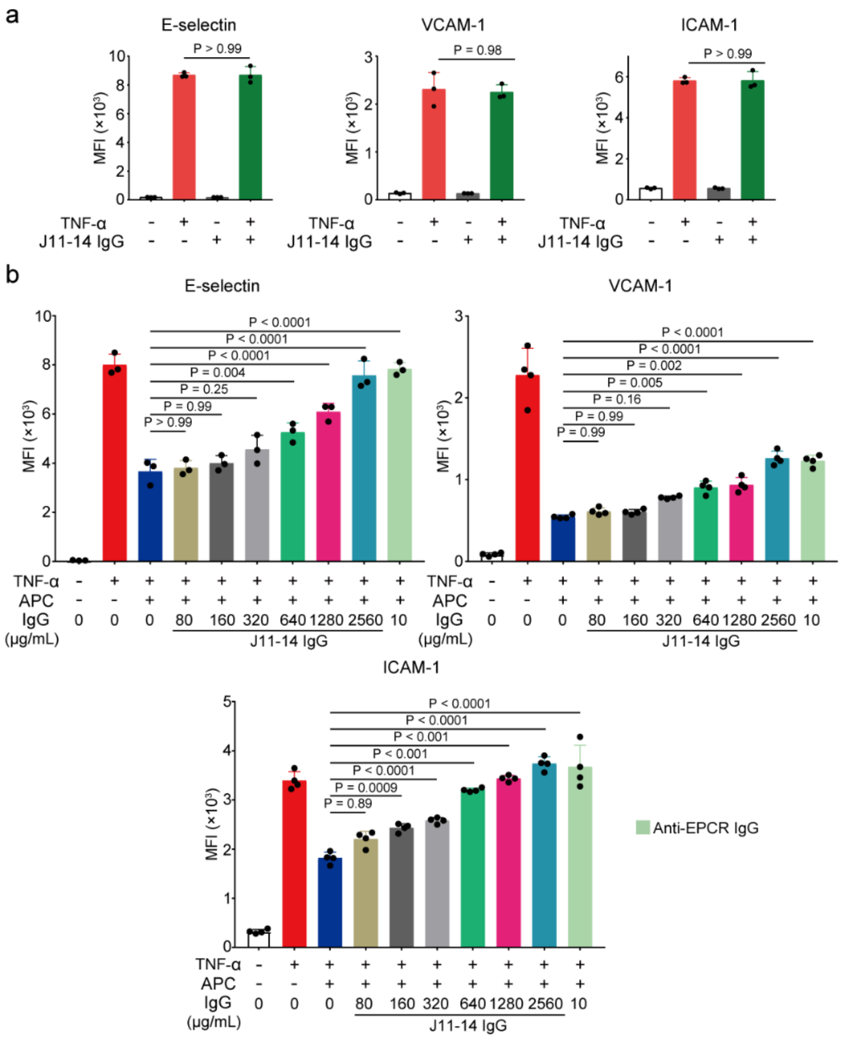

Supplementary Fig. 8 Dose-dependent blocking effect of anti-EPCR autoantibodies in Takayasu arteritis.

a HUVECs were first treated with or without 2.56 mg/mL anti-EPCR positive TAK AECA (J11-14) for 1 hour. After the removal of supernatant, cells were treated without APC for 13 hours and stimulated with or without 100 pg/mL of TNF-α for 5 hours. The expression of adhesion molecules was analyzed with flow cytometry (n = 3). **b** HUVECs were first treated with 10 μg/mL anti-EPCR commercial IgG or IgG from anti-EPCR positive TAK AECA (J11-14) for 1 hour at the indicated concentrations. After the removal of supernatant, cells were treated with or without 10 μg/mL APC for 13 hours and stimulated with 100 pg/mL of TNF-α for 5 hours. The expression of adhesion molecules was analyzed with flow cytometry (n = 3-4). Data are indicated as mean ± SD. At least three independent experiments were performed. Statistical analysis was performed using one-way ANOVA test followed by Tukey's post hoc test. MFI, mean fluorescent intensity.

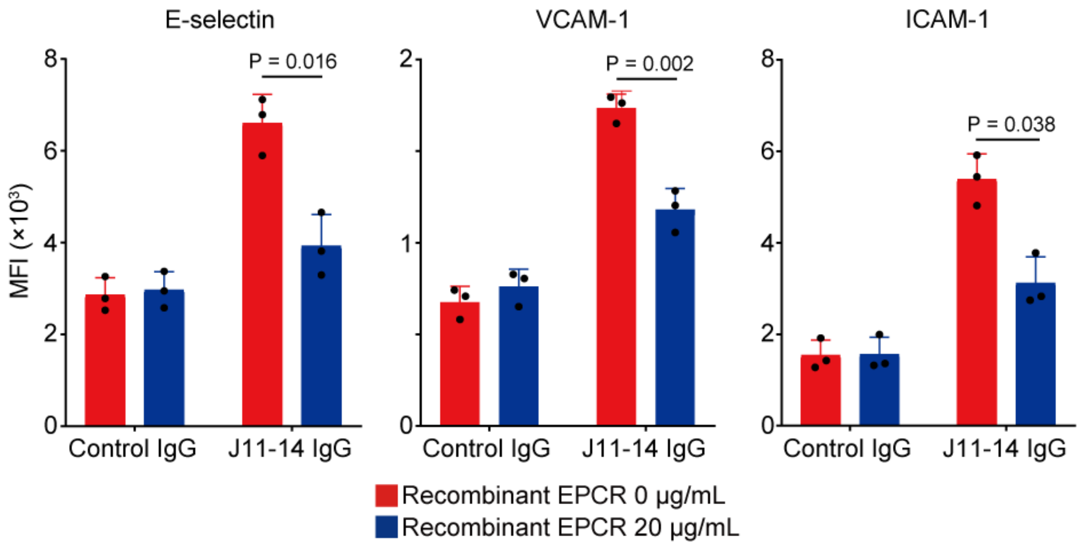

Supplementary Fig. 9 Soluble recombinant proteins reverse blocking effect of anti-EPCR autoantibodies in Takayasu arteritis.

HUVECs were first incubated with 2.56 mg/mL control IgG or 2.56 mg/mL IgG from anti-EPCR positive TAK AECA (J11-14) for 1 hour in the presence or absence of 20 μg/mL soluble recombinant EPCR. After the removal of supernatant, cells were treated with 10 μg/mL APC for 13 hours and stimulated with 100 pg/mL TNF-α for 5 hours. The expression of adhesion molecules was analyzed with flow cytometry (n = 3). Data are indicated as mean ± SD. At least three independent experiments were performed. Statistical analysis was performed using the paired *t* test. MFI represents mean fluorescent intensity.

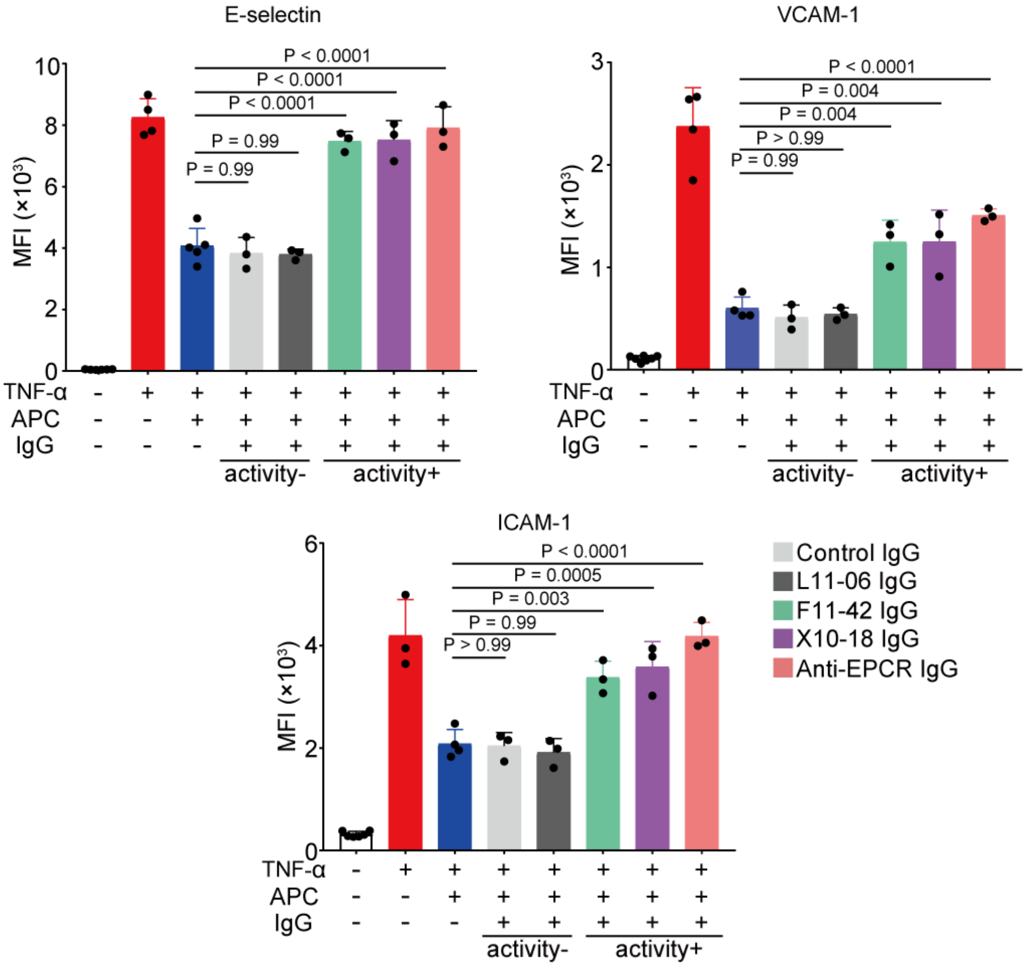

Supplementary Fig. 10 Regulation of endothelial cell activation by anti-EPCR autoantibodies in Takayasu arteritis.

HUVECs were first treated with 10 μg/mL anti-EPCR commercial IgG, 2.56 mg/mL IgG from anti-EPCR negative TAK AECA (L11-06) or 2.56 mg/mL IgG from anti-EPCR positive TAK AECA (F11-42, X10-18) for 1 hour. After the removal of supernatant, cells were treated with or without 10 μg/mL APC for 13 hours and then stimulated with 100 pg/mL TNF-α for 5 hours. The expression of adhesion molecules was analyzed with flow cytometry (n = 3-7). Data are indicated as mean ± SD. At least three independent experiments were performed in all cases. Statistical analysis was performed using ANOVA test followed by Tukey's post hoc test. MFI represents mean fluorescent intensity. L11-06, F11-42, and X10-18 indicate the serum sample number. Activity shows anti-EPCR activity.

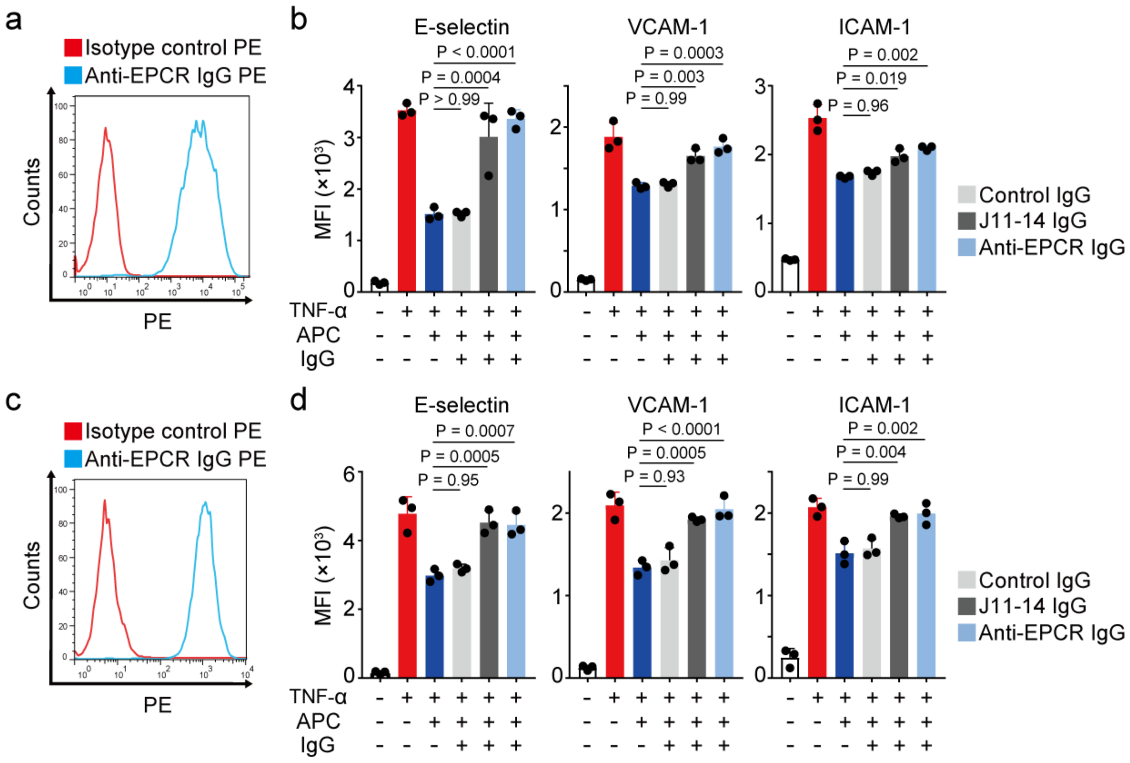

Supplementary Fig. 11 Anti-EPCR autoantibodies block the suppressive effect of APC in HAECs and HPAECs.

a, **c** Nonpermeabilized HAECs (**a**) or HPAECs (**c**) were stained with isotype control, or PE-conjugated anti-human EPCR IgG, and analyzed with flow cytometry. **b**, **d** HAECs (**b**) and HPAECs (**d**) were first treated with 10 µg/mL anti-EPCR commercial IgG or 2.56 mg/mL IgG from anti-EPCR positive TAK AECA (J11-14) for 1 hour. After the removal of supernatant, cells were treated with or without 10 µg/mL APC for 13 hours and then stimulated with 100 pg/mL TNF-α for 5 hours. The expression of adhesion molecules was analyzed with flow cytometry (n = 3). Data are indicated as mean ± SD. At least three independent experiments were performed in all cases. Statistical analysis was performed using ANOVA test followed by Tukey's post hoc test. MFI represents mean fluorescent intensity. J11-14 indicates the serum sample number.

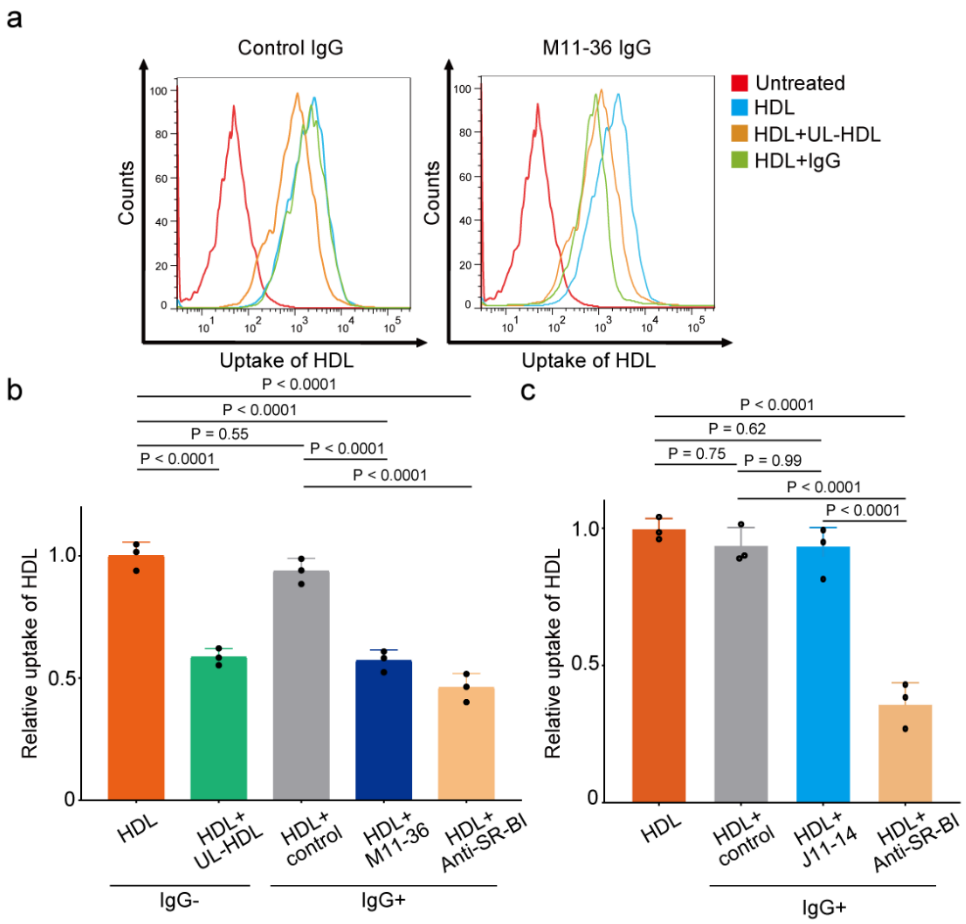

Supplementary Fig. 12 Anti-SR-BI autoantibodies in Takayasu arteritis inhibit uptake of HDL.

a, **b**, **c** HUVECs were starved for 4 hours followed by treatment with or without 20 µg unlabeled-HDL (UL-HDL), 2.56 mg/mL control IgG, 2.56 mg/mL IgG from anti-SR-BI positive TAK AECA (M11-36), or anti-EPCR positive TAK AECA (J11-14), or 10 µg/mL anti-SR-BI commercial IgG for 1 hour. After the removal of supernatant, cells were incubated with fluorescent HDL (10 µg) for additional 4 hours. Uptake of HDL was analyzed with flow cytometry (n = 3). Representative histograms (**a**) and summarized graph (n = 3) (**b**, **c**) are shown. Data are indicated as mean ± SD. At least three independent experiments were performed in all cases. Statistical analysis was performed using one-way ANOVA test followed by Tukey's post hoc test. M11-36 indicates the serum sample number.

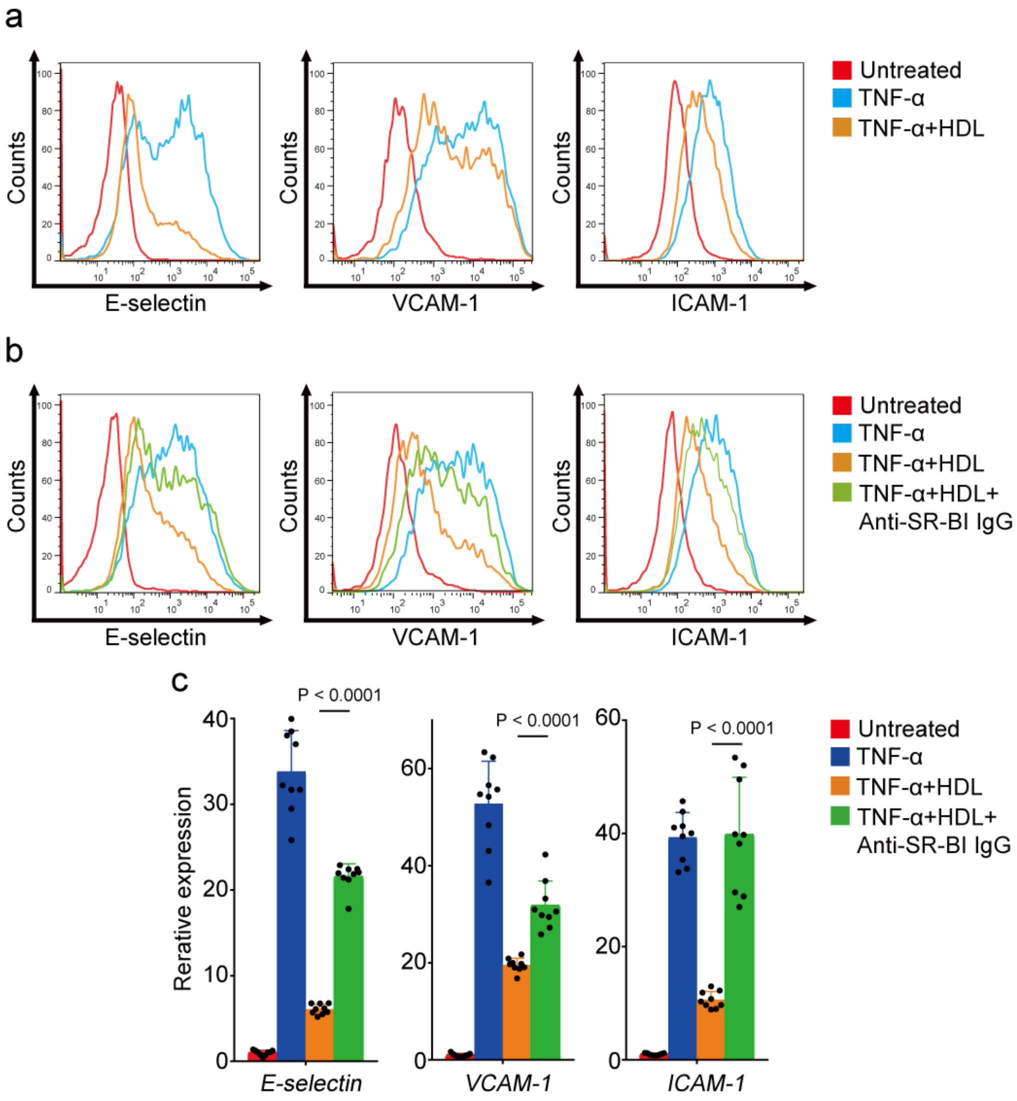

Supplementary Fig. 13 Anti-SR-BI commercial IgG blocks the suppressive effect of HDL upon upregulation of adhesion molecules.

a HUVECs were treated with or without 1 mg/mL HDL for 16 hours and then stimulated with 100 pg/mL TNF-α for 5 hours. The expression of adhesion molecules was analyzed with flow cytometry. **b**, **c** HUVECs were first treated with or without 10 μg/mL anti-SR-BI commercial IgG for 1 hour. After the removal of supernatant, cells were treated with or without HDL and stimulated with TNF-α as described. The expression of protein and mRNA of adhesion molecules was analyzed with flow cytometry (**b**) and quantitative PCR (n = 9) (**c**), respectively. Data are indicated as mean ± SD. At least three independent experiments were performed in all cases. Statistical analysis was performed using one-way ANOVA test followed by Tukey's post hoc test.

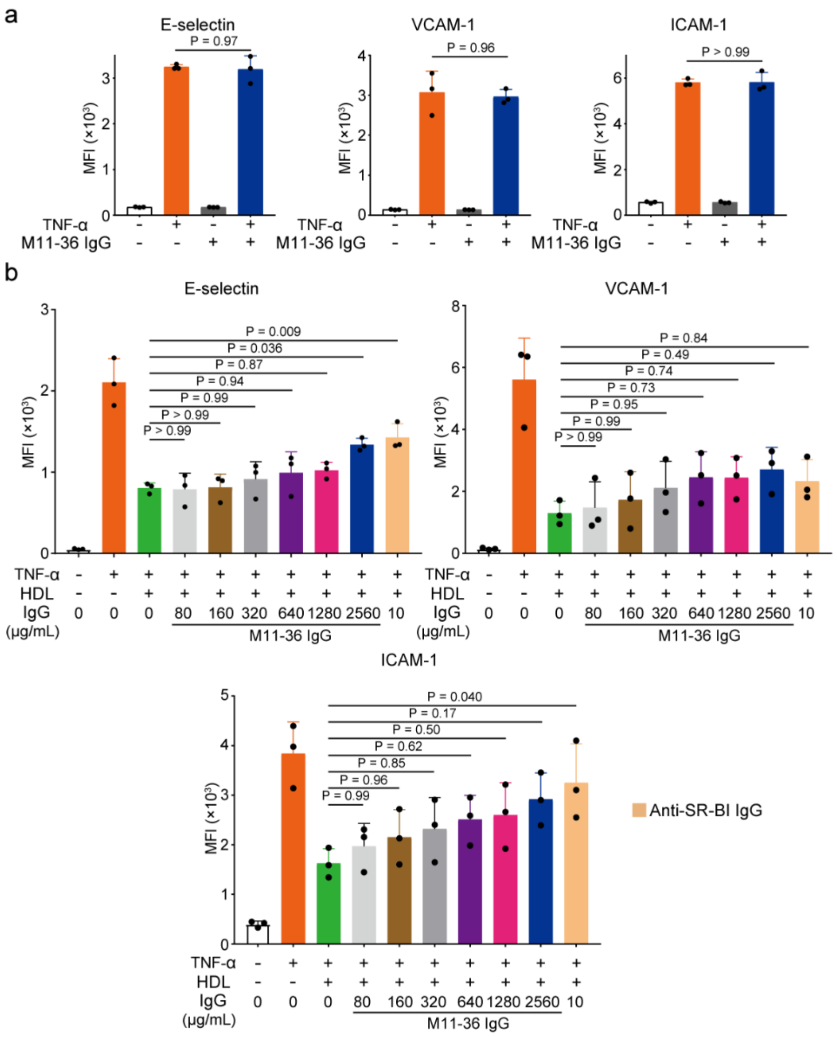

Supplementary Fig. 14 Dose-dependent blocking effect of anti-SR-BI autoantibodies in Takayasu arteritis.

a HUVECs were first treated with or without 2.56 mg/mL anti-SR-BI positive TAK AECA (M11-36) for 1 hour. After the removal of supernatant, cells were treated without HDL for 16 hours and stimulated with or without 100 pg/mL of TNF-α for 5 hours. The expression of adhesion molecules was analyzed with flow cytometry (n = 3). **b** HUVECs were first treated with 10 μg/mL anti-SR-BI commercial IgG or IgG from anti-SR-BI positive TAK AECA (M11-36) for 1 hour at the indicated concentrations. After the removal of supernatant, cells were treated with or without 1 mg/mL HDL for 16 hours and then stimulated with 100 pg/mL of TNF-α for 5 hours. The expression of adhesion molecules was analyzed with flow cytometry (n = 3). Data are indicated as mean ± SD. At least three independent experiments were performed in all cases. Statistical analysis was performed using one-way ANOVA test followed by Tukey's post hoc test. MFI represents mean fluorescent intensity. M11-36 indicates the serum sample number.

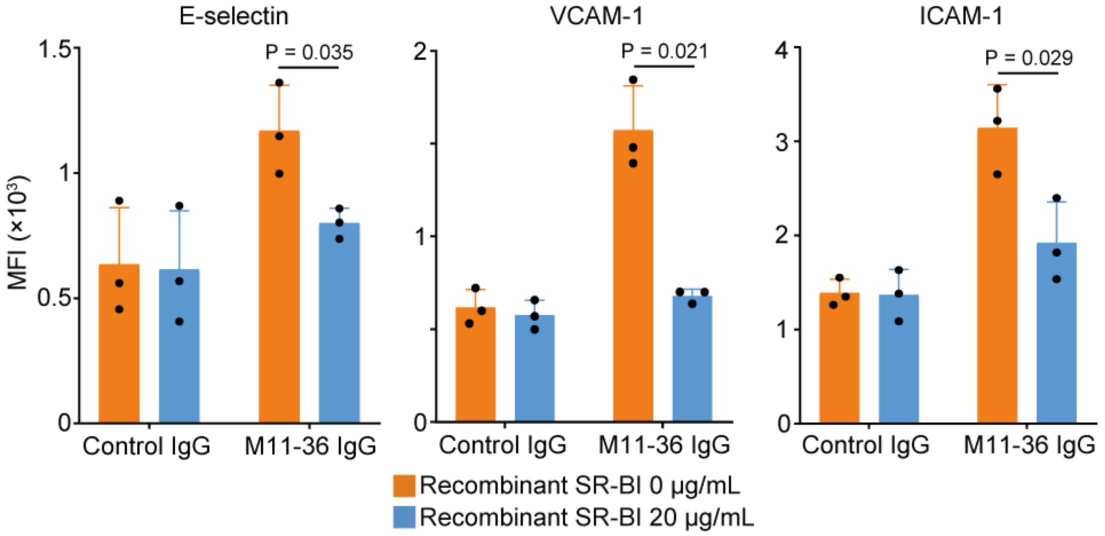

Supplementary Fig. 15 Soluble recombinant proteins reverse blocking effect of anti-SR-BI autoantibodies in Takayasu arteritis.

HUVECs were first incubated with 2.56 mg/mL control IgG or 2.56 mg/mL IgG from anti-SR-BI positive TAK AECA (M11-36) for 1 hour in the presence or absence of 20 μg/mL soluble recombinant SR-BI. After the removal of supernatant, cells were treated with or without 1 mg/mL HDL for 16 hours and stimulated with 100 pg/mL TNF-α for 5 hours. The expression of adhesion molecules was analyzed with flow cytometry (n = 3). Data are indicated as mean ± SD. At least three independent experiments were performed in all cases. Statistical analysis was performed using the paired *t* test. MFI represents mean fluorescent intensity. M11-36 indicates the serum sample number.

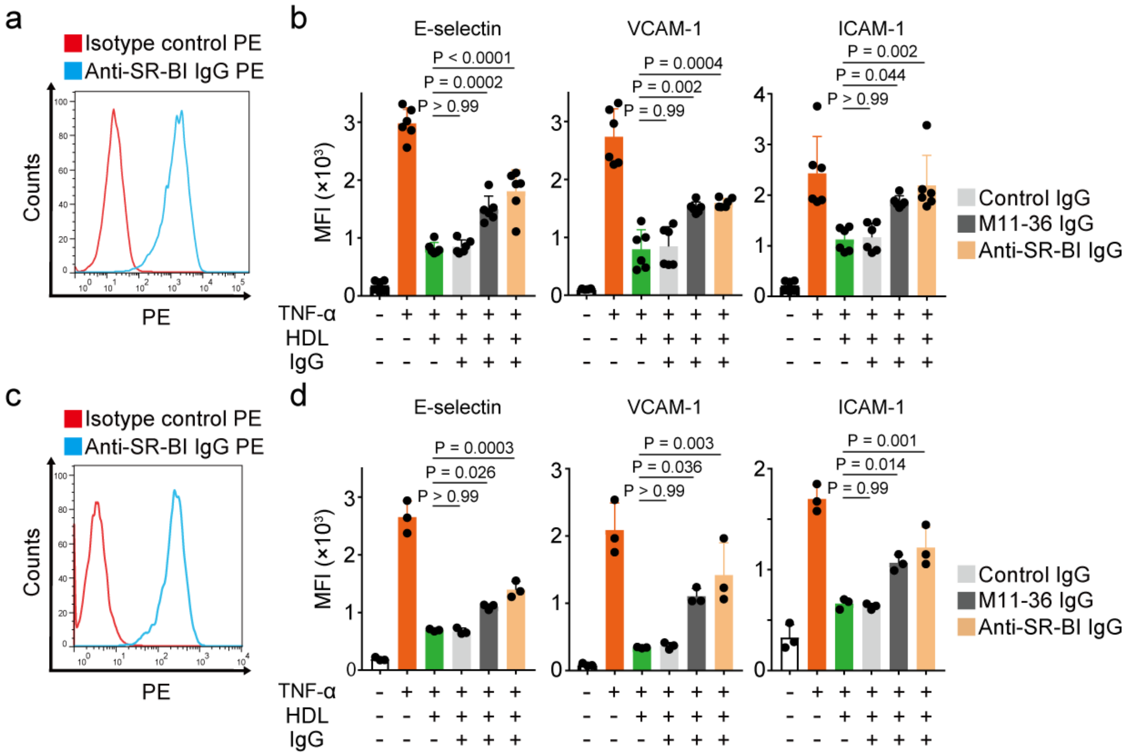

Supplementary Fig. 16 Anti-SR-BI autoantibodies block the suppressive effect of HDL in HAECs and HPAECs.

a, **c** Nonpermeabilized HAECs (**a**) or HPAECs (**c**) were stained with isotype control, or PE-conjugated anti-human SR-BI IgG, and analyzed with flow cytometry. **b**, **d** HAECs (**b**, n = 6) and HPAECs (**d**, n = 3) were first treated with 10 μg/mL anti-SR-BI commercial IgG or 2.56 mg/mL IgG from anti-SR-BI positive TAK AECA (M11-36) for 1 hour at the indicated concentrations. After the removal of supernatant, cells were treated with or without 1 mg/mL HDL for 16 hours and then stimulated with 100 pg/mL of TNF-α for 5 hours. The expression of adhesion molecules was analyzed with flow cytometry. Data are indicated as mean ± SD. At least three independent experiments were performed in all cases. Statistical analysis was performed using one-way ANOVA test followed by Tukey's post hoc test. MFI represents mean fluorescent intensity. M11-36 indicates the serum sample number.

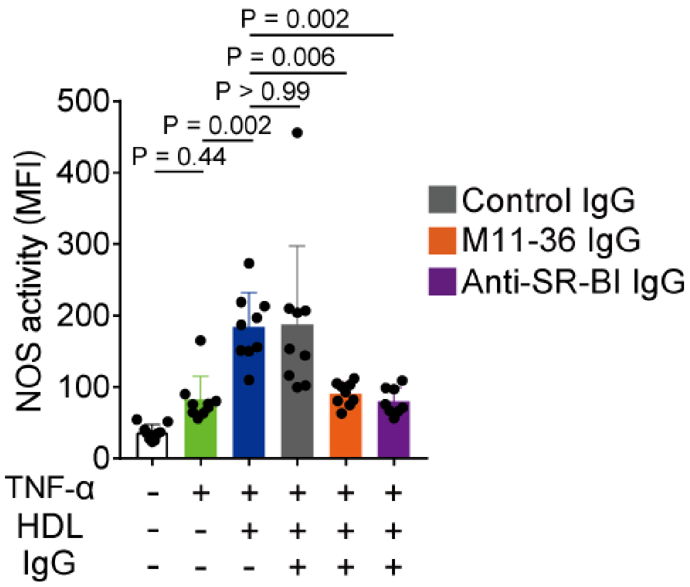

Supplementary Fig. 17 Anti-SR-BI autoantibodies block HDL induced NOS activity in HUVECs.

HUVECs were first treated with 10 μg/mL anti-SR-BI commercial IgG or 2.56 mg/mL IgG from anti-SR-BI positive TAK AECA (M11-36) for 1 hour. After the removal of supernatant, cells were treated with or without 1 mg/mL HDL for 16 hours and then stimulated with 100 pg/mL of TNF-α for 5 hours. After the removal of supernatant, cells were incubated with 1X Nitric Oxide Fluorometric Probe for 1 hour. The activity of NOS was analyzed with flow cytometry (n = 8-9). Data are indicated as mean ± SD. At least three independent experiments were performed in all cases. Statistical analysis was performed using one-way ANOVA test followed by Tukey's post hoc test. MFI represents mean fluorescent intensity. M11-36 indicates the serum sample number.

Supplementary Table 1. Baseline characteristics of 50 Takayasu arteritis patients with or without anti-EPCR or anti-SR-BI autoantibodies

	SR-BI+ EPCR- n = 17	SR-BI- EPCR+ n = 16	SR-BI- EPCR- n = 17
Age at onset (year)	41.2 ± 20.2	34.3 ± 14.1	35.5 ± 15.4
Sex (female)	15 (88.2%)	15 (93.8%)	16 (94.1%)
Duration until diagnosis (months)	6.0 (1–36)	3.5 (1-96)	4.0 (1–120)
Dissection	2 (11.8%)	0 (0%)	2 (11.8%)
Aneurysm	5 (29.4%)	4 (25.0%)	1 (5.9%)
Hypertension	9 (52.9%)	6 (37.5%)	9 (52.9%)
Relapse	8 (47.1%)	5 (31.3%)	9 (52.9%)
T-Chol (mg/dL)	178.1 ± 51,1	182.7 ± 46.4	196.4 ± 42.5
LDL (mg/dL)	99.7 ± 28.0	107.4 ± 36.1	109.7 ± 22.3
HDL (mg/dL)	58.1 ± 26.0	56.0 ± 19.2	61.8 ± 22.8
TG (mg/dL)	84.9 ± 38.3	96.4 ± 38.5	95.1 ± 79.6
Classification of affected vessel			
IIa	2 (11.8%)	5 (31.3%)	2 (11.8%)
IIb	2 (11.8%)	5 (31.3%)	6 (35.3%)
III	1 (5.9%)	0 (0%)	3 (17.7%)
IV	0 (0%)	0 (0%)	0 (0%)
Pulmonary artery	2 (11.8%)	2 (12.5%)	1 (5.9%)
Coronary artery	2 (11.8%)	2 (12.5%)	1 (5.9%)
Renal artery	6 (35.3%)	1 (6.3%)	5 (29.4%)
Ascending aorta	15 (88.2%)	14 (87.5%)	12 (70.6%)
Descending aorta	14 (82.4%)	9 (56.3%)	13 (76.5%)
Abdominal aorta	12 (70.6%)	4 (25.0%)	7 (41.2%)
CCA	14 (82.4%)	13 (81.3%)	12 (70.6%)
SCA	15 (88.2%)	13 (81.3%)	13 (76.5%)
BCA	8 (47.1%)	9 (56.3%)	7 (41.2%)

Values represent mean ± SD, median (minimum to maximum), or number (%). BCA, brachiocephalic artery; CCA, common carotid artery; HDL, high density lipoprotein; LDL, low density lipoprotein; SCA, subclavian artery; T-Chol, total cholesterol; TG, triglycerides.

Supplementary Table 2. Baseline characteristics of two Takayasu arteritis patients with anti-EPCR and anti-SR-BI autoantibody

	SR-BI+ EPCR+<br>n = 2
Age at onset (year)	37.5 ± 0.5
Sex (female)	2 (100%)
Duration until diagnosis (month)	28.5<br>(9-48)
Stroke	0 (0%)
Cardiovascular diseases	0 (0%)
Aortic regurgitation	0 (0%)
Dissection	0 (0%)
Aneurysm	0 (0%)
Hypertension	1 (50.0%)
Ulcerative colitis	1 (50.0%)
Surgical history	0 (0%)
Relapse	1 (50.0%)
CRP (mg/dL)	2.1 ± 1.5
ESR (mm/hour)	76.0 ± 49.7
T-Chol (mg/dL)	173.0 ± 43.8
LDL (mg/dL)	90.5 ± 36.1
HDL (mg/dL)	64.5 ± 12.0
TG (mg/dL)	101.5 ± 12.0
Classification of affected vessel	
I	0 (0%)
II	2 (100%)
IIa	1 (50%)
IIb	1 (50%)
III	0 (0%)
IV	0 (0%)
V	0 (0%)
Pulmonary artery	1 (50%)
Coronary artery	0 (0%)
Renal artery	0 (0%)
Ascending aorta	2 (100%)

Descending aorta	1 (50%)
Abdominal aorta	0 (0%)
CCA	2 (100%)
SCA	2 (100%)
BCA	2 (100%)

Values represent mean ± SD, median (minimum to maximum), or number (%). BCA, brachiocephalic artery; CCA, common carotid artery; CRP, C-reactive protein; ESR, erythrocyte sedimentation rate; HDL, high density lipoprotein; LDL, low density lipoprotein; SCA, subclavian artery; T-Chol, total cholesterol; TG, triglycerides.

Supplementary Method

Microarray analysis

Total RNA from cloned cells and unsorted cells was isolated and cDNA was generated, which was subjected to microarray analysis (GeneChip Human Genome U133 Plus 2.0 Array; Affymetrix, Santa Clara, CA, USA).

HDL uptake assay

HUVECs were starved for 4 hours followed by incubation with 10 μg fluorescently labeled HDL (BioVision, Milpitas, CA, USA) for 4 hours according to the manufacturer's instructions. The uptake of fluorescently labeled HDL was measured with flow cytometry. The effect of 2.56 mg/mL purified IgG, 10 μg/mL rabbit anti-human SR-BI antibody (PA5-29789, Abcam, 1:100, or NB400-113, Novus Biologicals, 1:100), or rat anti-human EPCR antibody [RCR-252] (ab81712, Abcam, 1:10) was examined, and further competed with 20 μg unlabeled-HDL (BioVision).

Detection of NOS activity

The OxiSelect^{TM} intracellular Nitric Oxide (NO) assay kit (Fluorometric) was purchased from Cell Biolabs. HUVECs were first incubated in 12-well culture plates (BD Biosciences) with or without IgG for 1 hour, followed by the addition of 1 mg/mL human HDL (Prospec Protein Specialist). Subsequently, HUVECs were stimulated by 100 pg/mL TNF-α (R&D Systems) for 5 hours. After removing medium from the wells, cells were washed 2 times with PBS, and incubated with serum free medium including 1X Nitric Oxide Fluorometric Probe (Cell Biolabs) in dark at 37°C for 1 hour. Then, fluorescence levels were measured with flow cytometry.
