# Supplementary material for: Identification of two major autoantigens negatively regulating endothelial activation in Takayasu arteritis
Source: Nat Commun. 2020 Mar 9;11:1253. doi: 10.1038/s41467-020-15088-0 (PMC7062749; doi:10.1038/s41467-020-15088-0)
Supplement: Supplementary file 3 — Reporting Summary [file 41467_2020_15088_MOESM3_ESM.pdf]

nature research

Corresponding author(s): Tsuyoshi Shirai

Last updated by author(s): 1/20/2020

Reporting Summary

Nature Research wishes to improve the reproducibility of the work that we publish. This form provides structure for consistency and transparency in reporting. For further information on Nature Research policies, see Authors & Referees and the Editorial Policy Checklist .

Statistics

For all statistical analyses, confirm that the following items are present in the figure legend, table legend, main text, or Methods section.

n/a	Confirmed	
☐	☒	The exact sample size (*n*) for each experimental group/condition, given as a discrete number and unit of measurement
☐	☒	A statement on whether measurements were taken from distinct samples or whether the same sample was measured repeatedly
☐	☒	The statistical test(s) used AND whether they are one- or two-sided<br>*Only common tests should be described solely by name; describe more complex techniques in the Methods section.*
☐	☒	A description of all covariates tested
☐	☒	A description of any assumptions or corrections, such as tests of normality and adjustment for multiple comparisons
☐	☒	A full description of the statistical parameters including central tendency (e.g. means) or other basic estimates (e.g. regression coefficient) AND variation (e.g. standard deviation) or associated estimates of uncertainty (e.g. confidence intervals)
☐	☒	For null hypothesis testing, the test statistic (e.g. *F*, *t*, *r*) with confidence intervals, effect sizes, degrees of freedom and *P* value noted<br>*Give P values as exact values whenever suitable.*
☐	☒	For Bayesian analysis, information on the choice of priors and Markov chain Monte Carlo settings
☐	☒	For hierarchical and complex designs, identification of the appropriate level for tests and full reporting of outcomes
☐	☒	Estimates of effect sizes (e.g. Cohen's *d*, Pearson's *r*), indicating how they were calculated

Our web collection on statistics for biologists contains articles on many of the points above.

Software and code

Policy information about availability of computer code

Data collection	Microsoft Excel 2016
Data analysis	GraphPad Prism 7.03 (GraphPad Software, La Jolla, CA, USA) and JMP ver.10.2 (SAS Institute, Japan)

For manuscripts utilizing custom algorithms or software that are central to the research but not yet described in published literature, software must be made available to editors/reviewers. We strongly encourage code deposition in a community repository (e.g. GitHub). See the Nature Research guidelines for submitting code & software for further information.

Data

Policy information about availability of data

All manuscripts must include a data availability statement. This statement should provide the following information, where applicable:

- Accession codes, unique identifiers, or web links for publicly available datasets
- A list of figures that have associated raw data
- A description of any restrictions on data availability

No datasets were generated or analysed during the current study

Field-specific reporting

Please select the one below that is the best fit for your research. If you are not sure, read the appropriate sections before making your selection.

☒ Life sciences ☐ Behavioural & social sciences ☐ Ecological, evolutionary & environmental sciences

For a reference copy of the document with all sections, see nature.com/documents/nr-reporting-summary-flat.pdf

Life sciences study design

All studies must disclose on these points even when the disclosure is negative.

Sample size	No-sample-size calculation was performed
Data exclusions	No data were excluded
Replication	All attempts at replication were successful
Randomization	This is not relevant to our study because this report is not prospective study.
Blinding	This is not relevant to our study because this report is not prospective study.

Reporting for specific materials, systems and methods

We require information from authors about some types of materials, experimental systems and methods used in many studies. Here, indicate whether each material, system or method listed is relevant to your study. If you are not sure if a list item applies to your research, read the appropriate section before selecting a response.

Materials & experimental systems

n/a	Involved in the study	
☐	☒	Antibodies
☐	☒	Eukaryotic cell lines
☒	☐	Palaeontology
☒	☐	Animals and other organisms
☐	☒	Human research participants
☒	☐	Clinical data

Methods

n/a	Involved in the study	
☒	☐	ChIP-seq
☐	☒	Flow cytometry
☒	☐	MRI-based neuroimaging

Antibodies

Antibodies used

All antibodies utilized for flow cytometry are described below:
FITC-conjugated goat anti-human IgG (Fab specific) (Sigma-Aldrich, catalog number: F5512, lot number: 046M4839V), PE-conjugated goat F(ab')2 anti-human IgG-Fc (Abcam, catalog number: ab98596, lot number: GR251065-2), PE-conjugated anti-CD201 (EPCR) antibody (BioLegend, catalog number: 351903, clone: RCR-401, lot number: B235674), rat anti-EPCR/CD201 antibody [RCR-252] (Abcam, catalog number: ab81712, lot number: GR3190270-1), PE-conjugated goat F(ab')2 anti-rat IgG-Fc (Abcam, catalog number: ab6259, lot number: GR116172-8), PE-conjugated mouse anti-human CD36L1 (SCARB1, SR-BI) antibody (BioLegend, catalog number: 363203, clone: m1B9, lot number:B227103), rabbit anti-human SR-BI antibody (Abcam, catalog number: PA5-29789), rabbit anti-SR-BI antibody (Novus Biologicals, catalog number: NB400-113), Scavenger receptor class B member 1 Antibody (Bioss Antibodies, catalog number: bs-1186R), PE-conjugated goat anti-rabbit IgG (Abcam, catalog number: ab72465, lot number: GR194665-18), PE-conjugated mouse anti-CD62E antibody (BioLegend, catalog number: 336008, clone: HAE-1f, lot number: B127523), PE/Cy5-conjugated mouse anti-CD106 antibody (BioLegend, catalog number: 305808, clone: STA, lot number: B211234), PE/Cy7-conjugated mouse anti-CD106 antibody (Novus Biologicals, catalog number: NBP-47864PECY7), Pacific Blue-conjugated mouse anti-CD54 antibody (BioLegend, catalog number: 322716, clone: HCD54, lot number: B239935), FITC-conjugated mouse anti-human CD3 antibody (BD Biosciences, catalog number: 555339,clone: HIT3a, lot number: 7095651), Pacific Blue-conjugated mouse anti-human CD4 antibody (BD Biosciences, catalog number: 558116, clone: RPA-T4, lot number: 6224744), APC-conjugated mouse anti-human IL-17A antibody (Biolegend, catalog number: 512333, clone: BL168, lot number: 7110887), APC/Cy7-conjugated mouse anti-human IL-17A antibody (BioLegend, catalog number: 512319, clone: BL168, lot number: B238373).

All antibodies utilized for western blotting are described below:
mouse anti-EPCR antibody (R&D Systems, catalog number: MAB22451, clone: 304519), rabbit anti-SR-BI antibody (Novus Biologicals, catalog number: NB400-104), mouse anti-α-tubulin antibody (Sigma-Aldrich, catalog number: 05-829, clone: DM1A), IRDye 800CW-conjugated goat anti-human IgG (H + L) (LI-COR Biosciences, catalog number: 926-32232), IRDye 800CW-conjugated goat anti-mouse IgG (LI-COR Biosciences, catalog number: 926-32210), IRDye 680-conjugated goat anti-rabbit IgG (LI-COR Biosciences, catalog number: 926-32221).

All antibodies utilized for immunohistochemistory are described below:
rabbit anti-human SR-BI antibody (Abcam, catalog number: PA5-29789), EPCR recombinant rabbit monoclonal antibody (Invitrogen, catalog number: MA5-29505)

Validation

Validation data are based on the manufacturer's website, data sheet, and relevant articles.

Eukaryotic cell lines

Policy information about cell lines

Cell line source(s)	Human umbilical vein endothelial cells (HUVECs), human aortic endothelial cells (HAECs), human pulmonary artery endothelial cells (HPAEC), and respective cell culture medium was purchased from Lonza (Basel, Switzerland). Rat myeloma cells, YB2/0, were purchased from American Type Culture Collection (Manassas, VA, USA). Plat-E packaging cells were purchased from Cell Biolabs (San Diego, CA, USA).
Authentication	Cell lines were purchased from companies described above.
Mycoplasma contamination	Cell lines were negative for mycoplasma contamination.
Commonly misidentified lines (See ICLAC register)	None

Human research participants

Policy information about studies involving human research participants

Population characteristics	Three hundred twenty five patients with collagen diseases were enrolled: 80, TAK; 10, giant cell arteritis; and 235, other collagen diseases. All the patients were diagnosed according to the respective criteria for classification. Seventy-nine age- and sex-matched healthy donors were enrolled as the control group.
Recruitment	We recruited patients who visited our department.
Ethics oversight	Tohoku University

Note that full information on the approval of the study protocol must also be provided in the manuscript.

Flow Cytometry

Plots

Confirm that:

- [x] The axis labels state the marker and fluorochrome used (e.g. CD4-FITC).
- [x] The axis scales are clearly visible. Include numbers along axes only for bottom left plot of group (a 'group' is an analysis of identical markers).
- [x] All plots are contour plots with outliers or pseudocolor plots.
- [x] A numerical value for number of cells or percentage (with statistics) is provided.

Methodology

Sample preparation	Human peripheral blood mononuclear cells (PBMC) were isolated by density gradient centrifugation using Ficoll-Paque PLUS (GE Healthcare, Uppsala, Sweden). Naïve CD4+ T cells were purified by negative selection using EasySep Human Naïve CD4+ T cell Isolation Kit (STEMCELL Technologies, Vancouver, BC, Canada).<br>Regarding Th17 differentiation, Purified naïve CD4+ T cells were cultured with Dynabeads human T-Activator CD3/CD28 (Invitrogen) (1:1 ratio of beads to cells), 10 ng/mL IL-6 (BD Biosciences), 10 ng/mL IL-1β (BD Biosciences), 10 ng/mL IL-23 (R&D Systems), 10 ng/mL TGF-β1 (R&D Systems), 10 μg/mL purified NA/LE mouse anti-human IFN-γ antibody (BD Biosciences), and 10 μg/mL purified NA/LE rat anti-human IL-4 antibody (BD Biosciences). The Th17 cells were stimulated with 50 ng/mL phorbol 12-myristate 13-acetate (PMA, Sigma-Aldrich) and 1 μg/mL ionomycin (Sigma-Aldrich) in the presence of GolgiStop (BD Biosciences) for 6 hours at day 7. Cells were fixed with Cytofix/Cytoperm Fixation and Permeabilization Solution Kit (BD Biosciences).
Instrument	Fluorescence intensity was measured using BD LSR Fortessa or FACS Canto II (Becton Dickinson, Franklin Lakes, NJ, USA). Cell sorting was performed using BD FACS Aria II (Becton Dickinson).
Software	FlowJo Software (Tree Star, Ashland, OR, USA)
Cell population abundance	The purity was confirmed by flow cytometry.
Gating strategy	Cell population was selected by FSC/SSC, and their fluorescence were assessed.

- [x] Tick this box to confirm that a figure exemplifying the gating strategy is provided in the Supplementary Information.
